# Changes in the soil organic carbon balance on China’s cropland during the last two decades of the 20^th^ century

**DOI:** 10.1038/s41598-017-07237-1

**Published:** 2017-08-02

**Authors:** F. Zhang, Z. Wang, S. Glidden, Y. P. Wu, L. Tang, Q. Y. Liu, C. S. Li, S. Frolking

**Affiliations:** 1 Department of Environmental Science and Technology, School of Human Settlements and Civil Engineering, Xi’an Jiaotong University, Xi’an, 710049 China; 2 State Key Laboratory of Loess and Quaternary Geology, IEE, CAS, Xi’an, 710075 China; 3 Institute for the Study of Earth, Oceans, and Space, University of New Hampshire, Durham, NH 03824 USA; 4 Ankang University, Ankang, 725000 China

## Abstract

Agro-ecosystems play an important role in regulating global changes caused by greenhouse gas emissions. Restoration of soil organic carbon (SOC) in agricultural soils can not only improve soil quality but also influence climate change and agronomic productivity. With about half of its land area under agricultural use, China exhibits vast potential for carbon (C) sequestration that needs to be researched. Chinese cropland has experienced SOC change over the past century. The study of SOC dynamics under different bioclimatic conditions and cropping systems can help us to better understand this historical change, current status, the impacts of bioclimatic conditions on SOC and future trends. We used a simulation based on historical statistical data to analyze the C balance of Chinese croplands during the 1980s and 1990s, taking into account soil, climate and agricultural management. Nationwide, 77.6% of the national arable land is considered to be in good condition. Appropriate farm management practices should be adopted to improve the poor C balance of the remaining 22.4% of cropland to promote C sequestration.

## Introduction

As a result of the global effects of anthropogenic activities^1^, such as the sharp increase in atmospheric C pools^2–4^, that greatly impact climate change^5^, people are considering feasible and effective ways to mitigate the impacts of these activities. Terrestrial ecosystems are thought to play an important regulatory role in the environment. Approximately 7–12% of carbon dioxide (CO_2_) emissions from anthropogenic sources are absorbed by terrestrial ecosystems^6^, and the annual C exchange through terrestrial ecosystems can reach 45 GtC (1 Gt = 10^15^g)^7^.

Agricultural soils contribute substantially to the terrestrial C pool^8^. The restoration of SOC in farmland soils has the capability to not only improve soil quality^9^ but also influence C emissions^10–12^ and increase agronomic productivity^13^. Furthermore, the change in SOC on farmland is directly affected by management practices and other farming conditions^14, 15^. The SOC in agro-ecosystems includes the C stock that is most closely related to human activities.

In China, approximately half of the land area in agriculture, which accounts for 10.7% of the world’s cropland (FAO, 2009; www.fao.org/statistics/en). The cropland in China exhibits vast C sequestration potential^16–18^. Studies based on soil surveys and regional simulations indicate that from the 1930s to the 1980s, there was a slight decrease in nationwide SOC storage in China^19, 20^. Extensive farm management is considered the major cause of continued SOC loss over this period^17^. The Chinese government began to promote conservation tillage and better management practices, including irrigation, fertilizer application and the use of crop residues, beginning in the 1990s. Since the late 1970s, the mechanization of water-saving irrigation has increased at an average annual rate of 9%, and the effective irrigation area increased by 12% from the 1980s to the 1990s^21^. Among the adopted farming reforms, changes in the ratios of chemical/organic fertilizers and crop residues were important improvements in agricultural technology in China in the second half of the last century. The use of machinery for straw crushing and returning increased at an average annual rate of 20.5% from 1990 to 2008^21^. The rapid increase in fertilizer use greatly increased food production and alleviated the pressure from the large human population in China. The 6.2% annual increase in fertilization from 1981 to 2000 increased the annual growth of crop production by 20%^21^ (www.fao.org/statistics/en). These management measures began to reverse the decline in SOC, and SOC began to accumulate due to the reform of agricultural technology in the 1990s^22^. Studies have shown that the decrease in cropland SOC in China gradually slowed after the 1990s and that SOC has begun to gradually increase^22, 23^.

Many studies on SOC changes have been conducted in China^16, 20, 22, 24, 25^; however, most of these studies have been based at the administrative level. Therefore, they have not addressed the effects of bioclimatic conditions on SOC. Studies have shown that the changes in SOC on Chinese cropland have been influenced by the climate and crop management^18^. Therefore, research into SOC change in different types of agro-ecosystems, such as in the midwestern dry-farming area of China, can be used to evaluate differences in bioclimatic conditions and cropping systems and their effects, thereby contributing to our understanding of the impacts of cropland management on SOC. In this study, we compiled data on SOC changes to analyze the regional C balance of Chinese cropland and its dynamics based on actual reproduction according to the DNDC (denitrification-decomposition model) scenario during the 1980s and into the 1990s.

## Results

### SOC stores on Chinese cropland

**Figure 1:**
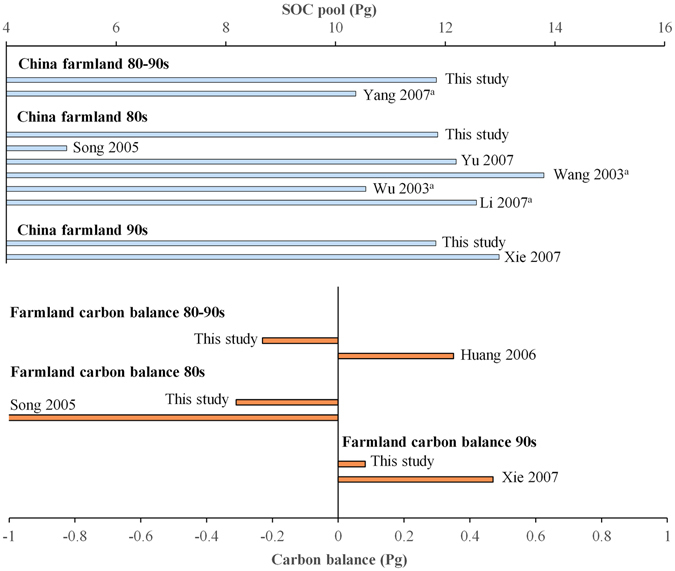
SOC balance on Chinese cropland. The figure was generated using Microsoft excel 2016 software provided by Microsoft office. ^a^Calculated based on a depth of 1 meter and 15% (Li & Zhao, 2001; Yu *et al*., 2007) of the total national soil carbon stored in agricultural soil.

The average SOC stock (0–50 cm) during the period from 1981 to 2000 was estimated to be 11.84 PgC for 93.74 Mha of cropland in China, which is consistent with a previously reported estimate of approximately 10.37 PgC (Fig. 1)^26^. In the 1980s, the national SOC stock was 11.86 PgC, which is within the previously reported range of 10.55–13.8 PgC^20, 22, 27–29^. However, other studies have suggested that SOC during this period might have been very low^30^. In the 1990s, the national cropland SOC stock was 11.83 PgC, which is consistent with the value of 12.98 PgC indicated by Xie *et al*.^22^, whose estimates were based on a second soil survey. Overall, evaluations of cropland SOC in China performed by different researchers largely agree.

**Figure 2:**
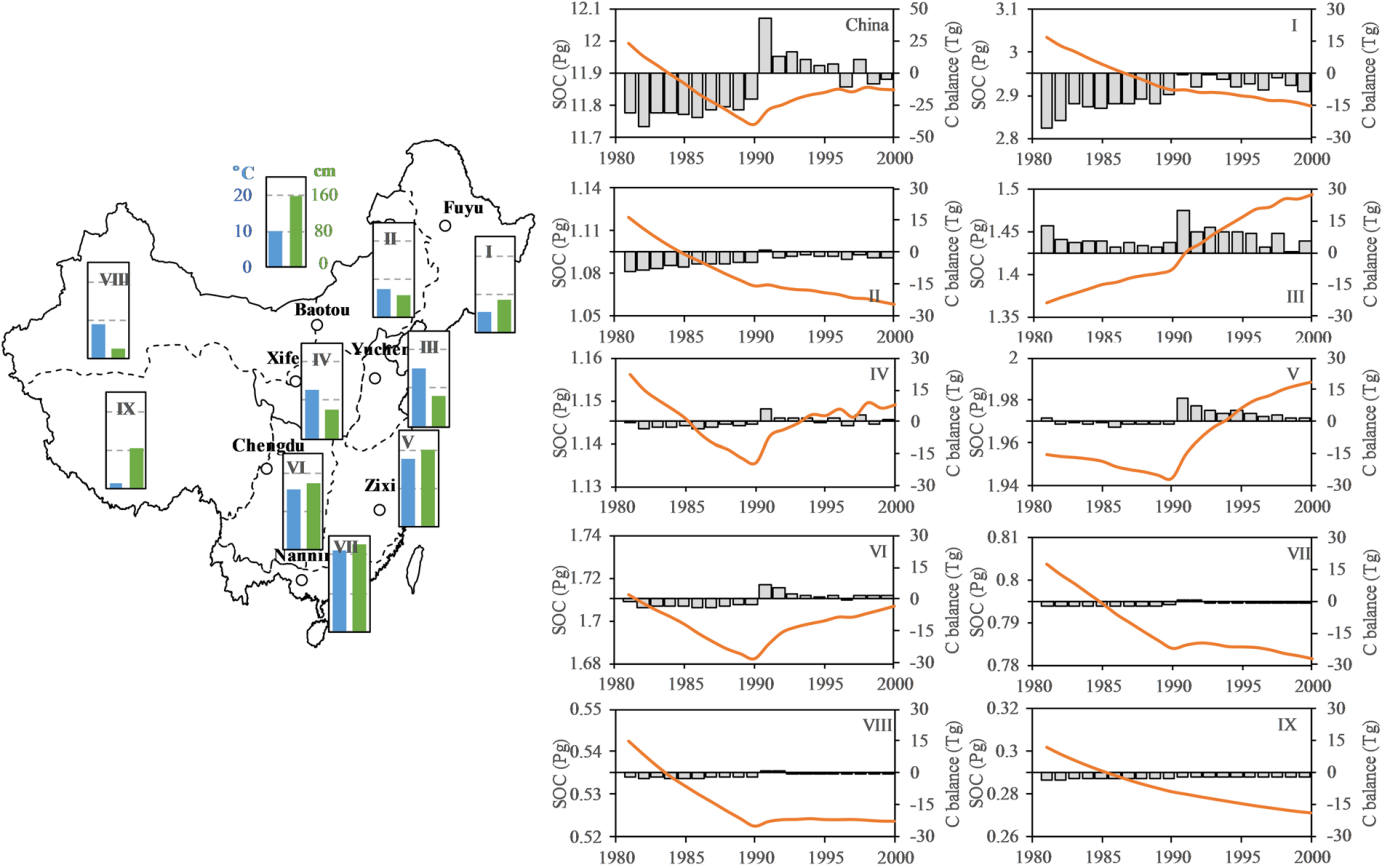
SOC change on cropland in China and in the 9 agricultural divisions. I, the northeastern region; II, Inner Mongolia and the area along the Great Wall; III, Huanghuai; IV, the Loess Plateau; V, the lower and middle reaches of the Yangtze River; VI, the southwestern region; VII, South China; VIII, Gansu-Xinjiang; and IX, the Qinghai-Tibetan area. The color of the map represents the extent of multiple cropping; the darker color indicates a higher proportion of multiple cropping. The floating bar graphs show the average annual temperature and precipitation in the region. The white points on the map of China show that the seven validation sites were located in different provinces and belong to different agro-ecosystem divisions. The figure was generated using Microsoft excel 2016 software provided by Microsoft office.

Previous studies addressing regional SOC have been based on administrative divisions (e.g., northeastern China, northern China, central China, southern China) due to their significant geographical differences. To better understand the changes in cropland SOC, however, farming types and characteristics should also be considered. In this study, the nation was divided into 9 agricultural divisions (Fig. 2)^31^. Overall, the national SOC stocks are higher in the southeast and lower in the northwest. SOC is generally high in the main crop production areas. The northeastern region exhibits the highest SOC, with an annual average SOC of 2.93 PgC, accounting for 25% of the national SOC. On the Loess Plateau, in Huanghuai, the lower and middle reaches of the Yangtze River and the southwestern area each possess 10–16% of the national SOC. SOC is lowest in Gansu Xinjiang and Qinghai Tibetan, each accounting for less than 5% of the national SOC. The northeastern region, the lower and middle reaches of the Yangtze River, and the southwestern area store 41% of the SOC and account for 47% of the cultivated acreage, representing the most important areas for cropland SOC storage in China. Based on the data^21^, the annual average rate of change in the crop planting area in 1981–2000 was 0.39%. Therefore, the change in cropland area impacts on SOC stock were not considered in this study.

### Carbon balance on Chinese cropland

No statistically significant differences in national SOC were found between the 1980s and 1990s or during the 20-year study period, indicating C neutrality of cropland SOC in China on an interdecadal scale. Piao *et al*.^25^ also stated that in the terrestrial ecosystems of China, cropland SOC is considered to be in equilibrium. However, the annual results of the present study indicate that differences exist in the change trend between the 1980s and 1990s. In the 1980s, the national SOC declined 0.02 PgC yr^−1^ and declined by 0.2 PgC overall (Fig. 2). Song *et al*.^30^ posited that the sharp decrease in SOC during this period was related primarily to long-term cultivation. In the 1990s, cropland SOC increased to 0.1 PgC, with an average increase of 0.01 PgCyr^−1^. This rate was lower than the increase in topsoil SOC of 0.047 PgC yr^−1^ on Chinese cropland from 1996 to 2006 reported by Xie *et al*.^22^. We speculate that this disparity may be related to the different soil depths used in the different studies.

Significant regional differences in the C balance were also found among agricultural districts over the 20-year period in this study. The Huanghuai area and the lower and middle reaches of the Yangtze River, located in eastern coastal areas, store 28% of the total SOC and account for 39% of the national arable land. Over the 20-year period, these areas exhibited a gradual increase in SOC (Fig. 2) and generally acted as a C sink. The Loess Plateau area in central China and the southwest region each showed a change similar to that observed for national SOC, which decreased in the 1980s and then increased in the 1990s (Fig. 2), with SOC being higher in 2000 than the average value for the 20-year period. These two regions store 24% of the total SOC and account for 27% of the national arable land, and they tend to be C neutral. SOC in the other regions showed a gradual decline over the 20-year period, with SOC being lower in 2000 than the 20-year average. The area presenting a decline in SOC was distributed mainly in the northeastern and northwestern regions of China, which store 48% of the total SOC on only 37% of the national arable land. The northeastern region is the major C source, with stocks representing approximately 50% of the total SOC in the C source area. Comparison of the z-scores for the agricultural districts clearly indicated three elements of SOC equilibrium: C sources, C sinks and C neutrality (Fig. 2).

**Figure 3:**
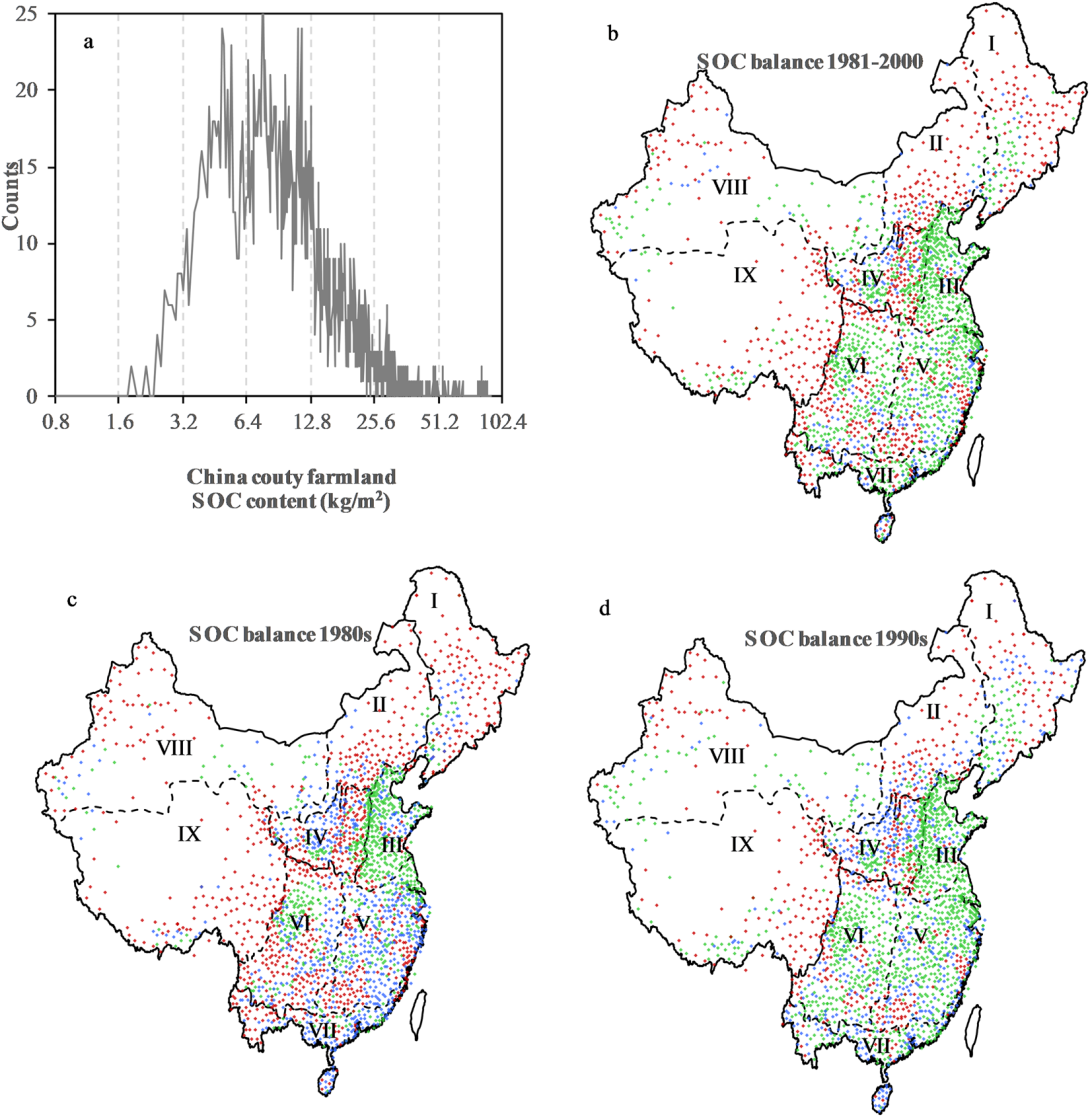
(**a**) SOC content of cropland in China. (**b**) SOC balance of cropland in China from 1981 to 2000. (**c**) SOC balance of cropland in the 1980s. (**d**) SOC balance of cropland in the 1990s. Green represents C sinks. Blue represents C-neutral areas. Red represents C sources. The figure was generated using Microsoft excel 2016 software provided by Microsoft office.

To further study the changes in SOC on Chinese cropland, statistics for the simulation results during the 20-year period in all county units were obtained based on the average national SOC content of 12.6 kgCm^−2^ (Fig. 3a). Areas with ΔSOC on a range of ±1% (±0.126 kgCm^−2^) were defined as representing C neutrality, whereas areas with ΔSOC >  + 0.126 kgCm^−2^ were defined as C sinks and those with ΔSOC < −0.126 kgCm^−2^ as C sources. Figure 3b indicates that the C source areas accounted for 29.75% of China’s arable land, which was distributed mainly in northeastern, Inner-Mongolian, northwestern regions, and in a small portion of the hilly and mountainous regions of south central China. Most of the cropland in mid-eastern China was shown to act as a C sink, accounting for 45.6% of the nation’s arable land. The C-neutral zone was distributed mainly in the central and southern regions and accounted for 24.65% of China’s arable land.

## Discussion

Cropland C equilibrium was directly correlated with cropland management. Phaeozem is a type of soil with a deep humus layer, uniform soil texture and high content of water-stable aggregates that develop under steppe meadows in temperate humid or semi-humid areas^32^. These soils are distributed mainly in humid or semi-humid areas with temperate and warm temperatures^33^ and are suitable for farming; the Phaeozem region in northeastern China exhibited the highest SOC content. However, intensive agricultural practices in areas with cropland exhibiting an approximately 50% slope^34^ and long slope length have caused Phaeozem to be vulnerable to water and erosion, resulting in a thinning of the Phaeozem^35^. The results indicate that in the northeast, the cropland along the Great Khingan Mountains, Lesser Khingan Mountains and Changbai Mountains lost the greatest amount of SOC, whereas the northeastern plains between the mountains showed significant recovery in the 1990s (Fig. 3). The drying of the northeastern region in recent decades^36^ and the large disaster area^37^ has also had important impacts on SOC losses, especially on sloped croplands.

Straw management could affect the SOC balance through C input and output. There are a wide variety of crops in China with rich straw resources. Crop residue are composed primarily of rice straw, wheat straw and corn stover, which accounted for 92% of the total residue from the main food crop^38^. In China, paddy rice, wheat and corn are planted mainly in the lower and middle reaches of the Yangtze River, the southwest, Huanghuai area and the northeastern region^21^. According to the conversion for crops and residues^38^, the aforementioned three main crop residues in these four regions accounted for 80% of the national residue yield. During the study period, the highest growth rate of residue production occured in the northeastern region and increased 5% annually^21^. A steady increase of C input through residue amendments effectively compensated for the carbon loss. The drop in SOC in the northeastern region eased during this period. The ΔSOC decreased from −13.5 TgC yr^−1^ in the 1980s to −3.9 TgC yr^−1^ in the 1990s and gradually exhibited stabilization.

Cropland SOC differs greatly across China. Significant C losses occurred mainly in the northeast and the southwest, and along the arid and semi-arid areas of the northern, northwestern and southwestern regions. In addition to varied soil parent materials and topography, the differences in climate and climatic fluctuation also contributed to SOC change. The coefficient of variation (CV) results demonstrated that the temporal variability of climatic factors in China varied greatly. Higher CVs were usually present in northern and western China where is colder and drier. This coincides with the regional pattern of SOC loss. The SOC content varied greatly in the northeastern region, Inner Mongolia, the area along the Great Wall, and in Gansu-Xinjiang. The CV values indicate strong climatic fluctuation (CV_IP_ , 0.18 ± 0.04; CV_IIP_ , 0.21 ± 0.04; CV_VIIIP_ , 0.23 ± 0.05) in these regions. This consistency indicated that heterogeneity in climate conditions and climatic fluctuation could affect the soil system.

The Qinghai-Tibetan plateau has unique geological and climatic conditions. Agriculture occurs mainly on the south plateau, which has a temperate climate where Ustic Cambisols and Gelic Cambisols dominate. The high humidity and thick humus layer lead to high SOC content in this region. Low CV values indicate that SOC change in this area may be more affected by the plateau topography and hydrothermal conditions.

Areas of double and triple cropping are distributed mainly in central and southeastern China, such as in Huanghuai, the lower and middle reaches of the Yangtze River, southwestern and southern China, where is warm and humid (Fig. 2). The three main crop straw in Huanghuai increased by 3.79% annually over 20 years^21^. Rich water and heat resources led to high C-input and a significant increase in SOC in the paddy soils in these regions.

The lower and middle reaches of the Yangtze River and the southwestern region cover a large part of southern China. Primary Ferrosols, enriched with iron and aluminum under warm conditions, are distributed in the lower and middle reaches of the Yangtze River and in the eastern part of southwestern China. Udic Ferrosols are distributed mainly in the low hilly mountainous areas in the lower and middle reaches of the Yangtze River, whereas in the southwest, Perudic Ferrosols are distributed mainly in the mountainous area of the Yunnan-Guizhou plateau^39^. The average annual temperature and precipitation was higher in the lower and middle reaches of the Yangtze River (~17.5 °C~1610 mm) compared to the levels in the southwestern region (~15.5 °C ~1400 mm) in both the 1980s and the 1990s. Relatively higher temperatures led to higher evaporation in the lower and middle reaches of the Yangtze River. Lower temperatures led to higher humidity and more leaching in the southwestern region, where there are also many Perudic Argosols. Therefore, organic matter accumulation would be easier in the lower and middle reaches of the Yangtze River than in the southwest. Because of the rough terrain and the large proportion of hilly areas, the Yunnan-Guizhou plateau is typically a barren soil area^39^, and some areas exhibited significant declines in SOC.

Crop residue in the lower and middle reaches of the Yangtze River and in the southwest accounted for 23.5% of the national total respectively. Because of the appropriate climate and geography, the lower and middle reaches of the Yangtze River have been the main rice-planting areas since the ancient times^40^. Stagnic Anthrosols were distributed mainly in this region, especially in the Yangtze River Delta. The formation of this soil type was strongly affected by human activities. The accumulation of humus could be promoted under flooding conditions in the process of rice cultivation and would be improved if the hydroponic duration were extended^39^. As a result, although there was still a large area of the Southwest that was a C source in the 1980s, the ΔSOC increased from −3.4 TgC yr^−1^ to 1.7 TgC yr^−1^ in the 1990s. By contrast, the lower and middle reaches of the Yangtze River exhibited a more significant transition from a C source to a C sink, with the ΔSOC increasing from −1.3 TgC yr^−1^ to 3.7 TgC yr^−1^ (Fig. 3).

The simulation indicated that changes in management practices, such as increasing C-inputs through straw amendments, affected the C balance across China in various ecological systems. Compared with their status in the 1980s, the source and sink status of arable soils across the nation differed greatly in the 1990s. The area acting as a C sink increased from 35.7% in the 1980s to 55.5% in the 1990s, and the C source area decreased from 37.6% to 21.9%. With the development of agricultural technology, the rates of SOC change slowed gradually, and the whole country approached a new balance after the first ten years of decline. In general, during the 20-year period, SOC on Chinese cropland tended toward balance and exhibited a slight increase. The cultivated soils in China changed from C sources to C sinks during this period. This change was most significant in southern China and was beginning to be observed in some areas in the western region (Fig. 3).

**Figure 4:**
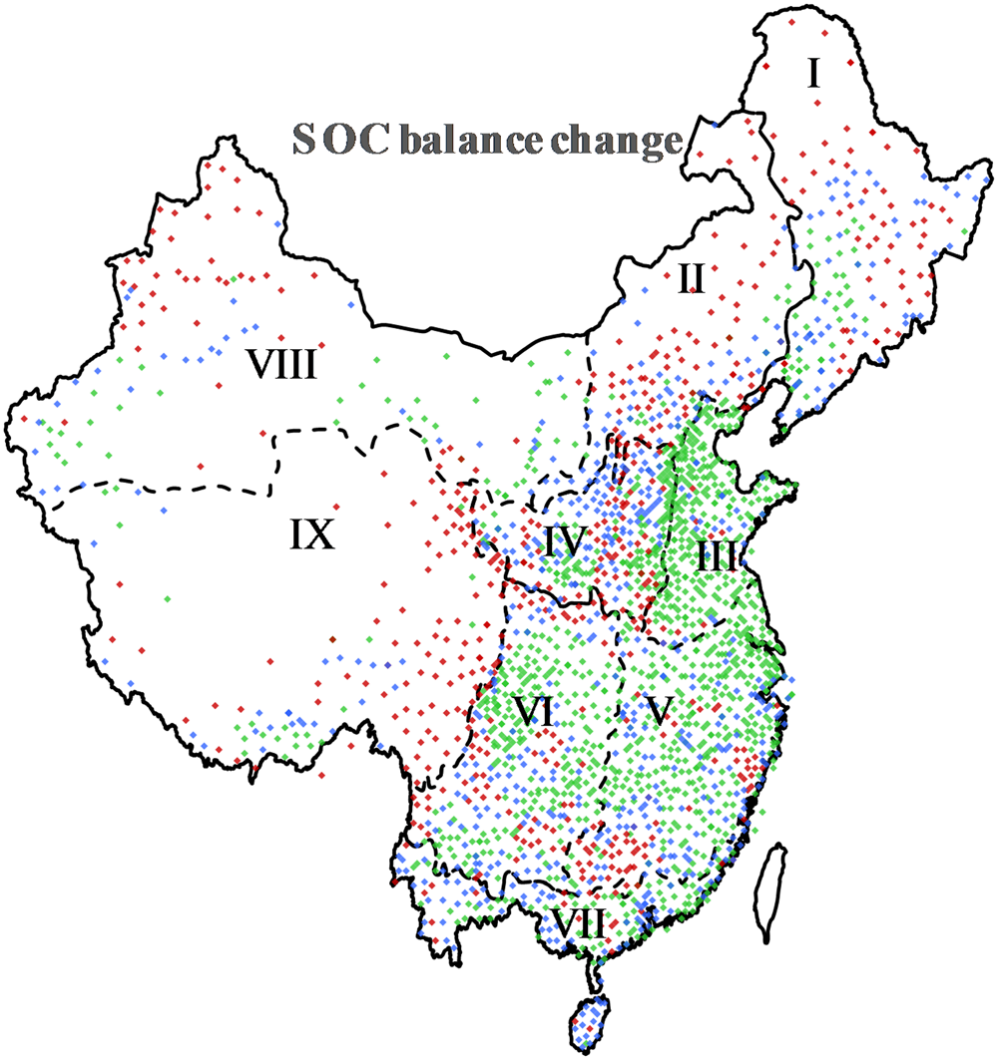
Type of turnover in SOC equilibrium state in all of the units in China. The turnover types were categorized based on the temporal trend of C balance on China’s cropland: healthy (was a sink or neutral in the 1980s and a sink in the 1990s), recovering (was a source or neutral in the 1980s and neutral in the 1990s), and degenerated (changed from a sink in the 1980s to neutral in the 1990s or was a source in both the 1980s and 1990s). The figure was generated using Microsoft excel 2016 software provided by Microsoft office.

Although Chinese cropland was found to be C neutral overall from 1981 to 2000, and to become a C sink after the 1990s, great differences in the types of SOC change were observed among the different regions during the 20-year period. In some valleys, such as the agricultural areas of the Loess Plateau, C loss occurred primarily in the 1980s, and the region acted as a significant C sink after the 1990s (Fig. 3). However, many regions exhibited a continued decline in SOC, such as the Great Khingan Mountains, the Lesser Khingan Mountains and the Changbai Mountains in northeastern China; the C equilibrium states of these areas differed greatly from those of the aforementioned valley areas and even more so from those areas that showed continued SOC increases during the 20-year period. The type of turnover in the C equilibrium state in all study areas during the 1980s and 1990s was classified as healthy, recovering or degenerated based on the temporal trend in C balance on Chinese cropland (Fig. 4). Figure 4 shows that the proportions of healthy, recovering and degenerated categories were 52.7%, 24.9% and 22.4%, respectively. Overall, China’s cropland is in good condition and has changed from a C source to a C sink.

Increasing vegetation has been acknowledged as an effective method of soil C sequestration. However, the complex relationship among soil, vegetation and climate forced us to consider the full range of environmental effects. For the whole nation, the CV of precipitation (CV_P_ , 0.16 ± 0.01) was significantly higher than the CV of temperature (CV_T_, 0.05 ± 0.1), indicating that the influence of climatic variability on agro-ecosystems in China from 1981 to 2000 was attributable primarily to changes in precipitation. Our analysis indicated that annual SOC could lose −0.15% to −1.76% without irrigation and increase 0.13% to 1.84% with irrigation, with a change rate that increased yearly. Furthermore, the increase in the SOC is slower on rain-fed croplands, especially in those vulnerable areas with low irrigation and high demand for mined groundwater^41^, e.g., the north and northwest. These are the fragile regions with significant C losses and strong climatic fluctuation. The trade-offs in water balance, vegetation growth and C sequestration must be taken into account^42^, particularly in these arid and semi-arid areas that have limited freshwater resources. Conservation tillage and water-saving irrigation patterns should be promoted in these areas. In order to improve the residue incorporation rate, intensive and mechanized planting methods would be the direction for future development.

## Methods

### Study material

DNDC was utilized in this study to reveal the SOC changes^43^. DNDC divides the soil into four C pools–residues, microbial biomass, humads and passive humus–based on the C conversion rates. Each of the SOC pools has a specific decomposition rate subject to temperature, moisture and N availability. Detailed management measures have been parameterized and linked to the various biogeochemical processes embedded in DNDC. The C distribution in each 10-cm layer was calculated based on the corresponding distribution coefficient. A surface profile of the top 50 cm of soil was calculated to analyze the farmland SOC changes. The regional changes were obtained by summing all of the SOC changes for all of the cropping systems within the 2473 counties during the 20-year period.

DNDC is driven by climate, soil, vegetation and anthropogenic activity. To extend the applications of DNDC to a national scale for China, we built a geographic information system (GIS) database, using county units to spatially aggregate differentiated information on climate, soil properties, vegetation and cropland management practices in the region. DNDC simulates 18 types of crop species in China. All of the crop species formed 18 single-, 23 double- and 5 triple-crop systems and one fallow system based on the climate, farming systems and customs of different areas. The crop and management data for the 2,473 counties in China were collected from provincial agricultural statistics (1981–2000, http://data.stats.gov.cn). Soil property data (i.e., bulk density, SOC content, texture and pH) were collected from the second national soil survey and the Chinese Soil Atlas (1:14,000,000)^44, 45^. Vegetation data (e.g., maximum yields, biomass partitions), fertilizer application rates and irrigated areas in the database were based on the Agricultural Economic Database (unpublished) of the Institute of Agricultural Resources and Regional Planning, Chinese Agricultural Sciences^46^, the agricultural census data and other published sources for China^47, 48^ (http://data.cma.gov.cn/data). Totals of 15% and 60% of the aboveground straw residues incorporated into the soil during the periods 1981–1990 and 1991–2000 respectively, were adopted for this study.

The daily weather data (e.g., maximum and minimum air temperatures, daily precipitation) were obtained from the Modern-Era Retrospective Analysis for Research and Application (MERRA)^49^. There are approximately 700 meteorological stations in China, which is far fewer than the 2,473 grid units of DNDC. In general, each climatic station was assigned to a cluster of units in the model based on their geographic location; therefore, some adjacent counties may share similar climate data in the simulation. Thus, as the density of meteorological data improves, the simulation accuracy can be expected to improve. This study utilized MERRA data to improve the simulation accuracy. As a result, 2,381 MERRA data were assigned to the 2,473 grid units to minimize the effect of climate variability.

Model initialization using average climatology for 100 years showed a C stabilization tendency after approximately 50 years. No significant differences were shown when the same GIS database and climate conditions were incorporated into the simulation. The CV represents the ratio of the standard deviation (σ) to the mean (μ). The CVs of temperature (CV_T_) and precipitation (CV_P_) were calculated to compare the degree of variability in the 20-year time series of the daily temperature and precipitation in China.

### Long-term validation of SOC in China

**Figure 5:**
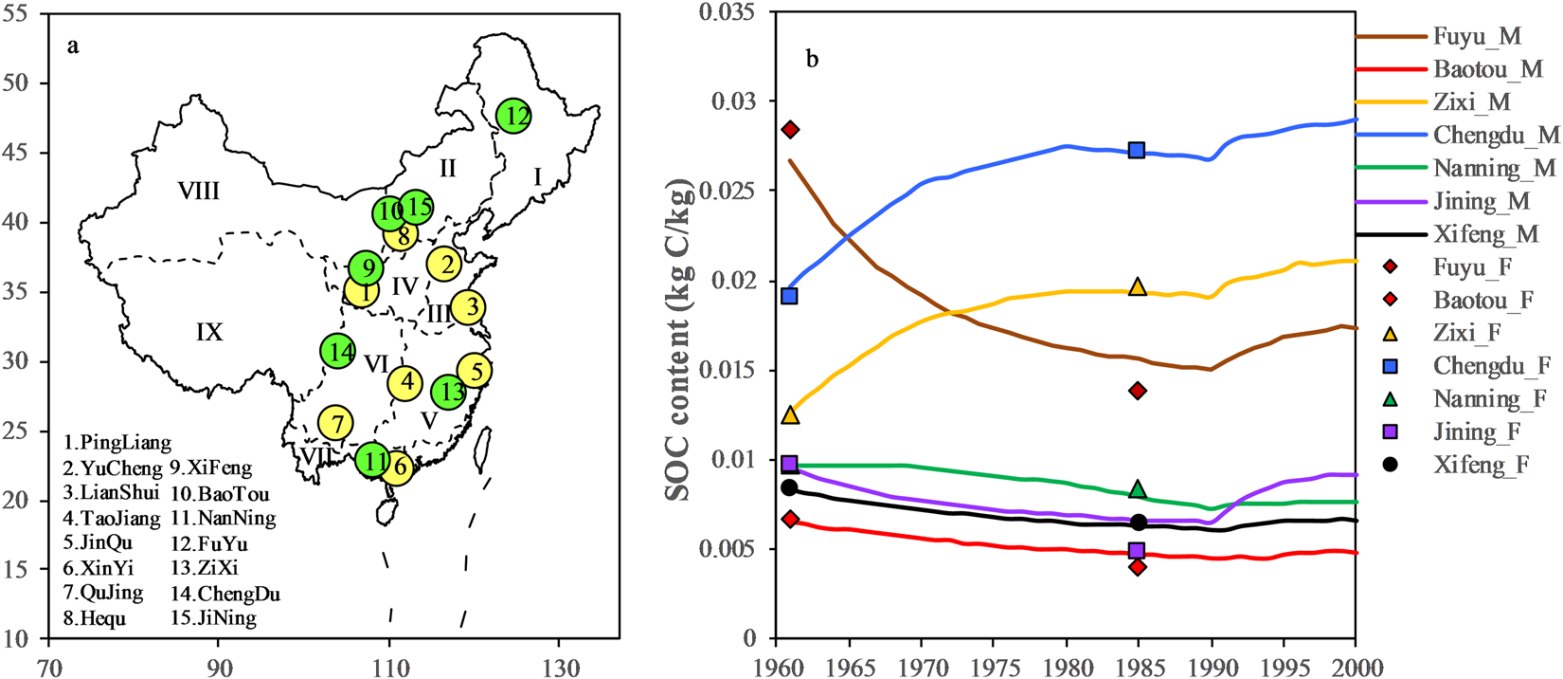
Long-term SOC validation of DNDC in China. The dots of the figure on the right represent field data from the two national soil surveys in China in the 1960s and 1980s. The line represents the simulation results. Sites 1 to 8 in the figure on the left are from Zhang *et al*. (2006, 2015). The figure was generated using Microsoft excel 2016 software provided by Microsoft office.

DNDC has been tested against data on SOC dynamics, greenhouse gas fluxes, and other parameters worldwide^18, 23, 50–52^. In this study, seven sites across different ecological regions in China, which were included in the two national soil surveys conducted in the 1960s and the 1980s, were chosen to further validate DNDC against long-term SOC dynamics (Fig. 5b).

The seven sites belong to different agro-ecosystem divisions (Fig. 2). The soil and vegetation data were sourced from the soil scientific database of China (www.soil.csdb.cn), national data (National Bureau of Statistics of China, data.stats.gov.cn) and a published monograph^45^. The climate data were obtained from meteorological stations in China. During the 25 years between the two national soil surveys, the SOC of Zixi in the middle reaches of the Yangtze River and Chengdu in southwestern China increased greatly (Fig. 5). The SOC exhibited a significant decline only in Fuyu which is located in northeastern China. Because of the relatively low background of SOC in Baotou, Xifeng, Jining and Nanning, there were no significant changes in these areas during the census. The validation indicated that the changes in SOC observed over the 40 years were associated with particular regional soil types and cropland management approaches.

### Model limitation

Model structure is acknowledged to be the primary source of uncertainty and can undermine predictions owing to incomplete specification of processes and parameters in an open system^53^. DNDC is a process-based valid model for agro-ecosystems. We determined that the performance of DNDC simulation in spots was good. When conducting an upscale simulation in large areas and over long time scales, data variability comes from spatial heterogeneity that would become one of the major sources of uncertainty. MERRA data, the Most Sensitive Factor (MSF) method and the Monte Carlo method were utilized to minimize uncertainty due to climate and soil input data. In particular, modeling based on the top 50 cm of soil may also lead to some uncertainty in the DNDC, although the soil differences appeared mainly in the topsoil and decreased with increasing depth.

## References

[1] Vitousek P (1997). Human domination of Earth’s ecosystems. Science.

[2] Friedli H, Loetscher HP, Oeschger H, Siegenthaler U, Stauffer B (1986). Ice core record of the 13C/12C ratio of the atmospheric CO_2_ in the past centuries. Nature.

[3] Keeling CD (2010). Atmospheric carbon dioxide variations at Mauna Loa Observatory, Hawaii. Tellus.

[4] Neftel A, Moor E, Oeschger H, Stauffer B (1985). Evidence from polar ice cores for the increase in atmospheric CO_2_ in the past two centuries. Nature.

[5] Barnola JM, Raynaud D, Korotkevich YS, Lorius C (1987). Vostok ice core provides 160,000-year record of atmospheric CO_2_. Nature.

[6] Janssens IA (2003). Europe’s terrestrial biosphere absorbs 7 to 12% of European anthropogenic CO_2_ emissions. Science.

[7] Esser, G. & Bouwman, A. F. Modelling global terrestrial sources and sinks of CO_2_ with special reference to soil organic matter. In *Soils And The* Greenhouse *Effect*. Edited by Bouwman, A. F. & Sons Ltd. 247–261 (1990).

[8] Cole CV (1993). Analysis of Agroecosystem Carbon Pools. Water Air Soil Poll.

[9] Tiessen H, Cuevas E, Chacon P (1994). The role of soil organic matter in sustaining soil fertility. Nature.

[10] Allen MR (2009). Warming caused by cumulative carbon emissions towards the trillionth tonne. Nature.

[11] Hoffert MI (2002). Advanced technology paths to global climate stability: Energy for a greenhouse planet. Science.

[12] Wise M (2009). Implications of Limiting CO_2_ Concentrations for Land Use and Energy. Science.

[13] Lal R (2006). Enhancing crop yields in the developing countries through restoration of the soil organic carbon pool in agricultural lands. Land Degrad Dev.

[14] Lal R (2004). Soil carbon sequestration impacts on global climate change and food security. Science.

[15] Leifeld J, Kogel-K I (2005). Soil organic matter fractions as early indicators for carbon stock changes under different land-use?. Geoderma.

[16] Huang Y, Sun W (2006). Changes in topsoil organic carbon of croplands in mainland China over the last two decades. Chinese Sci Bull.

[17] Lal R (2002). Soil carbon sequestration in China through agricultural intensification, and restoration of degraded and desertified ecosystems. Land Degrad Dev.

[18] Zhang F, Li C, Wang Z, Wu H (2006). Modeling impacts of management alternatives on soil carbon storage of farmland in Northwest China. Biogeosciences.

[19] Lindert PH (1999). The bad Earth? China’s soils and agricultural development since the 1930s. Economic Development and Cultural Change.

[20] Wang SQ, Tian HQ, Liu JY, Pan SF (2003). Pattern and change of soil organic carbon storage in China: 1960s-1980s. Tellus B.

[21] Ministry of Agriculture of P. R. China. *60 Years Agricultural Statistics Of New China*. Beijing: China Agriculture Press (2009).

[22] Xie ZB (2007). Soil organic carbon stocks in China and changes from 1980s to 2000s. Glob Change Biol.

[23] Zhang F (2015). Modeling impacts of management on farmland soil carbon dynamics along a climate gradient in Northwest China during 1981-2000. Ecol Model.

[24] Pan G, Li L, Wu L, Zhang X (2004). Storage and sequestration potential of topsoil organic carbon in China’s paddy soils. Glob Change Biol.

[25] Piao SL (2009). The carbon balance of terrestrial ecosystems in China. Nature.

[26] Yang Y, Mohammat A, Feng J, Zhou R, Fang J (2007). Storage, patterns and environmental controls of soil organic carbon in China. Biogeochemistry.

[27] Li ZP (2007). Assessment of soil organic and carbonate carbon storage in China. Geoderma.

[28] Wu H, Guo Z, Peng C (2003). Land use induced changes of organic carbon storage in soils of China. Glob Change Biol.

[29] Yu DS (2007). Regional patterns of soil organic carbon stocks in China. J Environ Manage.

[30] Song G, Li L, Pan G, Zhang Q (2005). Topsoil organic carbon storage of China and its loss by cultivation. Biogeochemistry.

[31] Comprehensive Agricultural Zoning of China Editorial Board, National agricultural zoning committee. *Comprehensive Agricultural Zoning Of China*. Beijing: China Agriculture Press (1981).

[32] Zhou, J. M. & Shen, R. F. Dictionary of Soil Science. Beijing: Science Press (2013).

[33] Ding, Y. H., Wang, S. W., Zheng, J. Y., Wang, H. J. & Yang, X. Q. *Climate In China*. Beijing: Science Press (2013).

[34] Zhang, D. & Gao, X. Z. Discussion on the comprehensive control measures for sloping farm land in black soil district. Jilin Water Resources **8**, 58–60 (2010).

[35] Xu, M. G., Liang, G. Q. & Zhang, F. D. *China’s Evolution Of Soil Fertility*. Beijing: China Agriculture Scientech Press (2006).

[36] Xie, A., Sun, Y. G. & Bai, R. H. Arid climate trend over Northeastern China and its response to global warming. Acta Geograhica Sinica **58**, 75–82 (2003).

[37] Ma JY, Xu YL, Pan J (2012). Analysis of agro-meteorological disasters tendency variation and the impacts on grain yield over northeast China. Chinese Journal Of Agrometeorology.

[38] Center of the national agricultural technology extension service. *Records Of Nutrients In Organic Fertilizer In China*. Beijing: China Agriculture Press (1999).

[39] Gong, Z. T., Huang, R. J. & Zhang, G. L. *Pedogeography Of China*. Beijing: Science Press (2014).

[40] Zhang YC (1982). Research of relations between climate change during history and rice plant area development in China. Chinese Science Bulletin.

[41] Grogan DS (2015). Quantifying the link between crop production and mined groundwater irrigation in China. Sci. Total. Environ..

[42] Jackson RB (2005). Trading water for carbon with biological sequestration. Science.

[43] Li CS, Frolking S, Frolking TA (1992). A Model of nitrous-oxide evolution from soil driven by rainfall events. 1. Model structure and sensitivity. J Geophys Res-Atmos.

[44] Institute of Soil Science, Academia Sinica. *The Soil Atlas Of China*. Beijing: Cartographic Publishing House (1986).

[45] National Soil Survey Office of China. *Soils In China*. Beijing: Agricultural Publishing House; vol. 1–6, 1993–1997.

[46] Tang H, Qiu J, Ranst EV, Li C (2006). Estimations of soil organic carbon storage in cropland of China based on DNDC model. Geoderma.

[47] Cui, D. C., Liu, H. S. & Min, J. R. *Atlas Of Major Agricultural Climate Resources Of China*. Beijing: Meteorological Press (1984).

[48] Liu, X. H. & Chen, F. *Farmring Systems In China*. Beijing: China Agriculture Press (2005).

[49] Rienecker MM (2011). MERRA: NASA’s modern-era retrospective analysis for research and applications. Journal of Climate.

[50] Cai ZC (2003). Field validation of the DNDC model for greenhouse gas emissions in East Asian cropping systems. Global Biogeochem Cy.

[51] Giltrap DL, Li C, Saggar S (2010). DNDC: A process-based model of greenhouse gas fluxes from agricultural soils. Agriculture, ecosystems & environment.

[52] Smith WN (2012). Crop residue removal effects on soil carbon: Measured and inter-model comparisons. Agriculture, Ecosystems & Environment.

[53] Oreskes, N. The role of quantitative models in science. in Models in Ecosystem Science (ed. Canham, C.D., Cole, J.J., Lauenroth, W.K.) Princeton: Princeton University Press, 13–31 (2003).

